# Supplementary material for: Third-wave cognitive behaviour therapies for weight management: systematic review and network meta-analysis protocol
Source: BMJ Open. 2018 Aug 1;8(7):e023425. doi: 10.1136/bmjopen-2018-023425 (PMC6074635; doi:10.1136/bmjopen-2018-023425)
Supplement: Supplementary file 1 [file bmjopen-2018-023425supp001.pdf]

Supplementary file: Medline search strategy

1	exp Obesity/
2	exp Overweight/
3	exp Body Weight/
4	exp Body Mass Index/
5	exp Waist Circumference/
6	exp Feeding Behavior/
7	exp Body Weight Changes/
8	exp Caloric Restriction/
9	exp Weight Loss/
10	obes*.mp.
11	(overweight or over-weight).mp.
12	(weight adj3 (body or chang* or loss* or maint* or manag* or control* or reduct*)).mp.
13	(food adj3 (intake or habit*)).mp.
14	(body mass index or bmi).mp.
15	body adj3 mass.mp.
16	(calori* adj3 (restrict* or restrain* or reduc*)).mp.
17	feeding adj3 behavio*.mp.
18	(diet* adj3 (restrict* or restrain* or reduc*)).mp.
19	(waist* adj3 circumferenc*).mp.
20	1 or 2 or 3 or 4 or 5 or 6 or 7 or 8 or 9 or 10 or 11 or 12 or 13 or 14 or 15 or 16 or 17 or 18
21	((3rd or third) adj3 wave).mp.
22	(acceptance* adj3 (commit* or mind* or base* or focus* or intervention* or therap* or treat*)).mp.
23	exp Mindfulness/
24	(mindful* or mind-ful*).mp.
25	(compassion* adj3 (mind* or base* or focus* or intervention* or therap* or treat*)).mp.

26	(behav* adj3 activation).mp.
27	((meta-cognit* or metacognit*) adj3 (mind* or base* or focus* or intervention* or therap* or treat*)).mp.
28	(dialectic* adj3 (behavio* or mind* or base* or focus* or intervention* or therap* or treat*)).mp.
29	(schema* adj3 (mind* or base* or focus* or intervention* or therap* or treat*)).mp.
30	(function* adj3 analyt*).mp.
31	(relation* adj3 frame*).mp.
32	20 or 21 or 22 or 23 or 24 or 25 or 26 or 27 or 28 or 29 or 30
33	20 and 32

Search terms 21 to 31 based upon Linardon et al.'s[18] search strategy. No database search restrictions will be applied.
