# Supplementary material for: Third-wave cognitive behaviour therapies for weight management: systematic review and network meta-analysis protocol
Source: BMJ Open. 2018 Aug 1;8(7):e023425. doi: 10.1136/bmjopen-2018-023425 (PMC6074635; doi:10.1136/bmjopen-2018-023425)
Supplement: Author's manuscript [file bmjopen-2018-023425.draft_revisions.pdf]

BMJ Open

BMJ Open is committed to open peer review. As part of this commitment we make the peer review history of every article we publish publicly available.

When an article is published we post the peer reviewers' comments and the authors' responses online. We also post the versions of the paper that were used during peer review. These are the versions that the peer review comments apply to.

The versions of the paper that follow are the versions that were submitted during the peer review process. They are not the versions of record or the final published versions. They should not be cited or distributed as the published version of this manuscript.

BMJ Open is an open access journal and the full, final, typeset and author-corrected version of record of the manuscript is available on our site with no access controls, subscription charges or pay-per-view fees (http://bmjopen.bmj.com).

If you have any questions on BMJ Open's open peer review process please email info.bmjopen@bmj.com

BMJ Open

Third-wave cognitive behaviour therapies for weight management: Systematic review and network meta-analysis protocol.

Journal:	*BMJ Open*
Manuscript ID	bmjopen-2018-023425
Article Type:	Protocol
Date Submitted by the Author:	05-Apr-2018
Complete List of Authors:	Lawlor, Emma; MRC Epidemiology Unit, University of Cambridge<br>Islam, Nazrul; MRC Epidemiology Unit, University of Cambridge<br>Griffin, Simon; The Primary Care Unit, Institute of Public Health<br>Hill, Andrew; University of Leeds, Division of Psychological and Social Medicine, School of Medicine<br>Hughes, Carly; Fakenham Medical Practice; University of East Anglia Faculty of Medicine and Health Sciences<br>Ahern, Amy; MRC Epidemiology Unit, University of Cambridge
Keywords:	Third-wave cognitive behaviour therapy, Weight management, Weight loss, Weight maintenance, Overweight, Obesity

SCHOLARONE™
Manuscripts

Third-wave cognitive behaviour therapies for weight management: Systematic review and network meta-analysis protocol.

Emma R Lawlor[1], Nazrul Islam[1], Simon J Griffin[1,2], Andrew J Hill[3], Carly Hughes[4,5], Amy L Ahern[1]

1. MRC Epidemiology Unit, University of Cambridge, Cambridge, England
2. Primary Care Unit, Institute of Public Health, University of Cambridge, Cambridge, England
3. Division of Psychological & Social Medicine, School of Medicine, University of Leeds, Leeds, United Kingdom
4. Fakenham Medical Practice, Norfolk, United Kingdom.
5. University of East Anglia, Norwich Medical School, Norwich, United Kingdom.

Corresponding author: Emma R Lawlor

Address: MRC Epidemiology Unit, University of Cambridge School of Clinical Medicine, Box 285 Institute of Metabolic Science, Cambridge Biomedical Campus, Cambridge, CB2 0QQ, United Kingdom.

Email: emma.lawlor@mrc-epid.cam.ac.uk

Telephone: +44 (0)1223 330315

Fax: +44 (0)1223 330316

Word count: 3704

For peer review only

ABSTRACT

Introduction: Behavioural and cognitive behavioural programmes are commonly used to assist with weight management, but there is considerable scope to improve their effectiveness, particularly in the longer term. Third-wave cognitive behaviour therapies (CBT) have this potential and are increasingly used. This systematic review will assess the effect of third-wave CBTs for weight management on weight, psychological and physical health outcomes in adults with overweight or obesity.

Methods and analysis: The systematic review will be reported according to the Preferred Reporting Items for Systematic Reviews and Meta-analyses guidance. We will include studies of any third-wave CBTs focusing on weight loss or weight maintenance for adults with a body mass index (BMI) ≥25kg/m^2. Eligible study designs will be randomised control trials (RCTs), non-randomised trials, prospective cohort and case series. Outcomes of interest will be body weight/BMI, psychological and physical health, and adherence. We will search the following databases from inception to 16th January 2018: MEDLINE, CINHAL, EMBASE, Cochrane database (CENTRAL), PsycINFO, AMED, ASSIA and Web of Science. The search strategy will be based on the concepts: 1) third-wave CBTs 2) overweight, obesity or weight management. No restrictions will be applied. We will search reference lists of relevant reviews and included articles. Two independent reviewers will screen articles for eligibility using a two-stage process. Two independent reviewers will extract data from studies, and assess risk of bias using the Critical Appraisal Skills Programme checklist. A random-effects network meta-analysis of outcomes, and sub-group analyses and meta-regression will be conducted, where the data permit. If not appropriate, a narrative synthesis will be undertaken.

For peer review only

Ethics and dissemination: Ethical approval is not required as no primary data will be collected. The completed systematic review will be disseminated in a peer-reviewed journal, presented at conferences and used to inform the development a weight management programme.

PROSPERO registration: CRD42018088255

Keywords: Third-wave cognitive behaviour therapy, Weight management, Weight loss, Weight maintenance, Overweight, Obesity

For peer review only

ARTICLE SUMMARY

Strengths and limitations of this study

- Different third wave CBT modalities and delivery methods will be distinguished enabling investigation into their comparative effectiveness.
- In addition to the direct treatment effects, indirect treatment effects will be analysed using a random-effects network meta-analysis.
- A comprehensive search strategy will be used with a large number of databases searched, no limitations applied and all prospective study designs included.
- A description of the intervention content, duration and delivery mode will be provided.
- It is anticipated that many papers will not provide sufficient details on all variables of interest and we will be reliant on communication with corresponding authors for additional information.

INTRODUCTION

Overweight and obesity is a major public health challenge, due to its high prevalence[1,2] and associations with reduced physical and psychological health,[3–5] as well as negative social and economic consequences.[6–8] Supporting people with overweight and obesity to achieve and maintain a healthier weight is an international priority. Most treatment approaches for overweight and obesity combine diet and physical activity advice with psychological support to make behavioural changes. Most commonly, this is a standalone treatment, although it is also an accompaniment to surgery or pharmacotherapy.[9]

There are a number of behavioural programmes that are effective for weight management in the short term, but there is substantial room for improvement in reach and effectiveness – particularly in terms of long term outcomes.[10–12] Whilst many people are able to apply behavioural strategies in order to lose weight, it is hard to sustain these in the face of obesogenic physical and social environments, the biological drive to maintain body weight, and the habitual nature of key behaviours.

One way that the effectiveness of behavioural programmes might be improved is to identify new strategies that target psychological processes associated with better long term weight loss and weight loss maintenance. Key amongst these is sustained motivation and the healthy and adaptive self-regulation of eating behaviour and emotions.[13–15] Third-wave cognitive behaviour therapies (CBTs) have been identified as a potentially useful treatment to address these important factors.

Third-wave CBTs

Third-wave CBTs have a number of core components that distinguish these approaches from first wave behaviour therapy and second wave cognitive behaviour

For peer review only

therapy; specifically an emphasis on openness, awareness, and action.[16] Types of third-wave CBTs include dialectical behavioural therapy (DBT), schema therapy (ST), acceptance and commitment therapy (ACT), acceptance-based behavioural treatment (ABBT), mindfulness-based cognitive behavioural treatment (MBCT) or compassion-focussed therapy (CFT).[16–18] These treatments have been associated with adaptive self-regulation and sustained motivation across a number of health domains, for example, in depression and addiction.[19–21]

There are a number of ways in which these treatments could support successful weight management. For example, fostering non-judgemental awareness of thoughts, feelings and behaviour, could enable people to reduce overeating and to limit the influence of internal and external cues. Encouraging patients to experience potentially aversive internal experiences (cravings, anxiety, behavioural fatigue) and to pursue behaviour that is congruent with their goals and values could support them in adhering to a weight management plan – even when weight loss has plateaued. Fostering a compassionate attitude toward the self could help prevent discouragement following minor lapses. Mindfulness and acceptance-based therapies have also been associated with improvements in psychological outcomes related to long term weight management, including self-regulation, dietary restraint, emotional eating, body satisfaction and mood.[22–26]

Evidence to date

Previous systematic reviews have investigated the effect of third-wave CBTs on weight management.[22–28]

Although these reviews have consistently reported improvements for obesity related eating behaviours and psychological outcomes, results for weight loss have been

For peer review only

mixed. Only three reviews of interventions in people with overweight and obesity included a meta-analysis of weight or BMI outcomes.[22,25,26] Rogers et al.'s[25] meta-analysis of eight studies reported a small but significant reduction in BMI, and the 16 studies included in Carrière et al.'s[22] meta-analysis reported a significant moderate effect on weight loss. However, no significant effect was found for BMI in Ruffault et al.'s[26] meta-analysis of nine studies. These differences may be attributable to variation in inclusion criteria and methods across the reviews.

In addition to mixed findings, there are a number of methodological limitations that constrain conclusions on the effectiveness of third-wave CBT for weight management. The majority of reviews utilised a small number of databases,[24–27] lacked a comprehensive search strategy,[26] and conducted their database searches during, or prior to 2016,[23,25–28] meaning that existing reviews will not have captured all relevant research. In addition, there has been an emphasis on mindfulness, with some reviews excluding other therapy types[22,23] and others using the term 'mindfulness' to encompass a range of third-wave CBTs. There has been a general failure in reviews to distinguish between different types of third-wave CBT or different methods of delivery, and detail on intervention content, delivery mode and intensity is lacking. This causes difficulties in assessing the comparative effectiveness of different approaches and limits their potential to inform future studies. Furthermore, little is known regarding participant adherence and attrition as only Olson and Emery[28] provided detail on this outcome. This would provide important insights into intervention fidelity and acceptability.

The present systematic review will provide a more comprehensive review of the range of third-wave CBTs that have been utilised for weight management. It will interrogate a large number of databases and include all prospective study designs,

For peer review only

while acknowledging the strength of randomised controlled trials. Importantly, it will distinguish between different treatment modalities and delivery methods, by pulling both the direct head-to-head comparisons reported in earlier studies, and the indirect and mixed effects within a random-effects network meta-analysis framework. Meta-regression methods will be applied to identify and/or adjust for potential source(s) of heterogeneity. While change in weight is the primary outcome, this review will also capture a range of physical and psychological health outcomes and will summarise data on intervention adherence. This review will provide important information with which to inform the development and refinement of third-wave CBTs for weight management.

Objectives

We aim to evaluate the effectiveness of third-wave CBTs for weight management in adults with overweight or obesity.

Primary objective

- To evaluate the effectiveness of third-wave CBTs for weight loss and weight loss maintenance in adults with overweight and obesity.

Secondary Objectives

- To evaluate the effect of third-wave CBTs for weight loss and weight loss maintenance on psychological and physical health outcomes in adults with overweight and obesity.
- To provide a detailed description of the content, duration, and delivery of interventions.

- To identify the intervention characteristics that are associated with better outcomes and adherence.

METHODS AND ANALYSIS

Eligibility criteria

Studies will be selected according to the criteria outlined below.

Study designs

We will include original research articles, theses and dissertations reporting randomised control trials (RCTs), non-RCTs, prospective cohort (PC) and case series studies that report an outcome measure pre- and post-intervention. No restrictions will be placed upon language, year of publication or publication status.

Participants

We will include studies of community dwelling adult human participants (aged ≥18 years) with overweight or obesity (BMI ≥ 25 kg/m^2). Participants must be seeking assistance with weight loss or weight loss maintenance. No further restrictions will be made on gender, age, recruitment method, or co-morbid conditions.

Interventions

Studies will be included if they evaluated a third-wave CBT for the purpose of weight loss or weight loss maintenance. In terms of defining third-wave CBTs, they must be described as using DBT, ST, ACT, ABBT, MBCT or CFT. As these approaches are sometimes delivered as part of a multi-component intervention, no restriction will be placed on the proportion of the intervention using the technique, although this may

For peer review only

be considered in secondary-analyses. Interventions will be of any duration or delivery mode. There will be no restriction placed on who delivers the intervention. If multiple arms are included in a study, any arm that meets the inclusion criteria will be included in the review. We will exclude studies that do not state that weight management is an aim of the intervention.

Comparators

For the meta-analysis, we will include studies with no comparator i.e., single-arm pre-post studies with no control arms. We will also compare third-wave CBTs to (i) no intervention or minimal intervention, and (ii) other behavioural programme(s), as reported in the studies.

Outcomes

The primary outcomes will be:

- Body weight
- BMI

The secondary outcomes will be:

- Stress
- Anxiety
- Depression
- Meta-cognition
- Eating attitudes
- Eating behaviours
- Body satisfaction

For peer review only

- Quality of life
- Blood pressure
- Lipids
- Glycaemia
- Adherence to treatment

Timing

We will include studies that report one or more of the primary or secondary outcome measures pre- and post-intervention. We will exclude studies in which the follow up measures are less than 12 weeks from baseline, because of the identified need to find new approaches to improve long-term weight loss and weight loss maintenance.

Setting

No laboratory based interventions will be included. All other settings are eligible for inclusion.

Language

There will be no language restrictions.

Information sources and search strategy

Electronic searches

We will search the following databases: CINHAL, MEDLINE, EMBASE, Cochrane database (CENTRAL), PsycINFO, AMED, ASSIA and Web of Science. No restrictions to the search strategies will be applied, and databases will be searched from inception to the 16th of January 2018. For studies published in a non-English

For peer review only

language, appropriate employees of the university will be contacted to request a translation.

To identify studies for inclusion in this review, detailed search strategies will be developed for each electronic database by ERL, who has previous experience conducting systematic reviews, with input from a medical librarian. Other members of the systematic review team will also be consulted to ensure appropriateness of terminology and that no terms have been overlooked. The search strategy will include a number of key word and subject heading searches relating to the concepts: (1) Third-wave CBTs AND (2) Overweight, obesity or weight management. Searches in the other databases will be based upon the MEDLINE search strategy (see supplementary additional file), with modification when appropriate to take into consideration database-specific terms. Search terms for the third-wave CBTs concept will be based upon a systematic review of third-wave CBTs for eating disorders by Linardon et al.[18]

Other search methods

To enhance literature saturation, we will manually search reference lists of all primary studies identified as eligible for inclusion in the review and previous relevant reviews. Authors of abstracts that were identified in the database searches will be contacted to identify whether the study findings have been published elsewhere or accepted for publication. A bibliography of the included studies will be circulated to the systematic review team to ensure no other studies they are aware of are excluded.

Study records

For peer review only

Data management and selection process

We will import results from the searches into a Microsoft Excel spreadsheet and duplicates will be removed. We will screen the study titles and abstracts to eliminate articles which clearly do not meet the inclusion criteria. There will be an initial piloting of this screening with an identical 10% of articles independently screened by two researchers to ensure consistency. If a high degree of disagreement occurs, the inclusion and exclusion criteria of the studies will be clarified through discussion with a third reviewer. Once piloting is completed, all article titles and abstracts will be independently screened by two researchers. We will obtain full text papers where titles and abstracts are deemed to be relevant or where eligibility is unclear. The obtained full text articles will then be independently screened by two researchers and their eligibility will be discussed to gain consensus. Where necessary, we will seek additional information from study authors to resolve any questions about eligibility. Reasons for exclusion of articles at the full text screening stage will be recorded. A third reviewer will resolve disagreements, if required. Reviewers will not be blinded to authors, institution or journal when screening articles. In the case of multiple articles pertaining to the same study, all articles will be included and then collated to make best use of the data. A Preferred Reporting Items for Systematic Reviews and Meta-Analyses (PRISMA)[29] flow-chart will be presented showing the process of study selection.

Data collection process

For studies that fulfil the inclusion criteria, we will extract data from articles onto a data collection form. This form will be based upon the Cochrane data extraction form,[30] the Consolidated Standards of Reporting Trials (CONSORT) 2010

For peer review only

statement[31] and the Template for Intervention Description and Replication (TIDieR) checklist.[32] This will ensure that an appropriate breadth and depth of detail will be captured. The data extraction form will be piloted on three articles before it is finalised. Data will be independently extracted by two researchers and any discrepancies will be resolved by a third reviewer. Data analysis will be conducted using statistical program Stata version 14.2 (StataCorp. 2015. *Stata Statistical Software: Release 14*. College Station, TX: StataCorp LP.).

Data items

Data to be extracted from the studies will include:

- General information (e.g. study authors, publication year, country, source of funding)
- Study aim
- Population description
- Study characteristics (e.g. study design, randomisation, blinding, allocation concealment)
- Participants (e.g. age, sex, race/ethnicity, socioeconomic status, diagnosed condition(s), recruitment methods, sample size, weight)
- Intervention characteristics (e.g. therapy type/content, mode of delivery, group or individual delivery, dose of intervention, duration of session, setting, profession who delivered the intervention, theoretical framework)
- Comparator intervention characteristics (e.g. therapy type/content, mode of delivery, group or individual delivery, dose of intervention, duration of session, setting, profession who delivered the intervention, theoretical framework)

For peer review only

- Outcomes (e.g. outcome(s) studied, whether self-reported or objectively measured, duration of follow-up, statistical analysis, intervention effect sizes,)
- Adherence and attrition (e.g. total number of participants at baseline and at follow-up measurements, reasons for attrition, attendance, adherence to intervention)

If studies provide data for multiple follow-up time points, data will be extracted for all time points. If multiple arms are included in a study, data from any arm that meets the inclusion criteria will be extracted.

Due to the objective of this study to provide detailed descriptions of the content of the interventions, attempts will be made to contact the corresponding author of the study to retrieve further information not provided in the article, or in related publications. Study authors will also be contacted if there are any uncertainties regarding the study or missing data. If there is no response, authors will receive two email reminders. Authors will be given a two month timeframe to reply to this request for further information.

Outcomes and prioritisation

The primary outcomes of interest are body weight and BMI as the aim of our review is to evaluate the effect of the third-wave CBTs on weight management. Body weight will be reported as kg. BMI will be defined as body weight (kg) divided by height (m) squared, and will be reported as kg/m^2. We will record whether these measurements have been objectively assessed or have been obtained through participant self-report or other means.

For peer review only

Secondary outcomes are stress, anxiety, depression, meta-cognition, eating attitudes, eating behaviours, body satisfaction, quality of life, blood pressure, lipids, glycaemia and adherence to the third-wave CBT.

Psychosocial outcomes that have been associated with successful weight management have been included in order to understand the potential of third wave CBTs to target these potential determinants of longer term weight control. Data for these outcomes will be extracted as reported in the study. It will be noted if a validated instrument has been used.

For blood pressure, data for systolic and diastolic blood pressure will be extracted separately, and it will be reported using the unit mmHg. For lipids, data will be extracted for total cholesterol, low-density lipoproteins (LDL) and high-density lipoproteins (HDL) and reported using the unit mmol/l. If the ratio of total cholesterol to HDL has been presented, if possible, this will converted into the separate outcomes for total cholesterol and HDL, or the study author will be contacted for raw data. Glycaemia may be reported as glycated haemoglobin ($HbA_{1c;}$ mmol/mol or %) or glucose (random, fasting or 2hr; mmol/l). These outcomes are important as they are closely associated with weight and are risk factors for a range of chronic diseases.

Information relevant to adherence to treatment will include number of sessions attended, length and number of times practicing skills, use of resources, and deviations from intervention instructions. Any information regarding participant attrition/retention, reasons for attrition and attendance at sessions will also be collected. This is important as it will enable investigation into the effect of intensity of

For peer review only

interventions on weight management, and also give insight into the acceptability of the intervention.

Risk of bias of individual studies

Two researchers will independently assess the included studies for risk of bias. Any discrepancies will be discussed with a third reviewer to gain consensus. We will pilot this approach with three studies. Risk of bias and methodological quality will be assessed in accordance with the appropriate Critical Appraisal Skills Programme (CASP) checklist.[33,34] It is felt that this choice of assessment would be most appropriate due to the study designs eligible for inclusion e.g. non-RCTs, PC and case series. Choice of CASP checklist will be dependent on the study design. The majority of criteria within the checklist will be answered with 'yes', 'no' or 'can't tell' responses. Based on these criteria, studies will be classified as being of 'high', 'low' or 'unclear' risk of bias. Any other potential sources of risk of bias not covered by the CASP checklist will be noted. Reviewers will not be blinded to study authors, journal or institution. Results will be presented in a summary table.

Meta-biases

To assess for selective outcome reporting, if a study protocol is available, the outcomes reported in the protocol and the article will be compared. If not available, the methods and results section of the article will be compared to check for any inconsistencies.

Data synthesis

Intervention comparisons: Direct

For peer review only

Standard pairwise random-effects meta-analysis will be conducted where the homogeneity of data and intervention permit, using Stata version 14.2. The head-to-head comparisons will be conducted on the outcome measures reported at specific time-points of follow-up (e.g., 3 months, 6 months, 12 months). Studies that have outcomes that fall between these time-points will be combined with the closest time-point. Mean differences (for continuous data) and odds ratio (for categorical data), and their 95% confidence intervals will be estimated and reported.

Intervention comparisons: Indirect and mixed

Random-effects network meta-analysis will be conducted to estimate the indirect and mixed effects using the Stata suite of commands for network meta-analysis, along with commands for visualisation and reporting of results.

Sensitivity analyses

Sensitivity analysis will be conducted to see the potential impact on the effect estimates of different study designs (e.g., RCTs, cohort studies) by restricting the analysis to specific study types and/or by excluding one study designs at a time.

In the case of studies with two or more eligible intervention arms, dependent on the similarity of the interventions, results may be combined or be split into different groups. If different measures have been used to assess the same outcome and are sufficiently similar, they may be pooled or harmonised. If the effect measures are reported in different scales, meta-analysis will use standardised mean differences (standardised by the baseline SD value).

Statistical heterogeneity

For peer review only

The statistical heterogeneity (the portion of the variability that cannot be attributed to random error) of the studies will be tested using the I^2 statistic along with its 95% CI. Forest plots showing the overlap of the confidence intervals will also be provided to enable visual inspection. For the network meta-analysis, we will also report a total I^2 statistic, and the heterogeneity variance parameter (τ^2) estimated from the network meta-analysis models.

Analysis of subgroups or subsets

If sufficient data are available, subgroup analysis will compare:

- Type of intervention (e.g. therapy type, duration, delivery, intensity)
- Comparator intervention (e.g. minimal intervention or other behavioural intervention)
- Health condition of participants (e.g. type 2 diabetes)
- Length of follow up (e.g. 12 weeks, 6 months, 12 months)
- Study design (e.g. RCT, quasi-RCTs, PC, case series)
- Study quality (e.g. risk of bias)

If sufficient data on important covariates are reported in the studies, meta-regression techniques will be applied to identify and/or adjust for potential sources of heterogeneity, if applicable.

Narrative analysis

If meta-analysis is determined not to be appropriate due to substantial heterogeneity of studies and outcome measures that cannot be pooled, a narrative synthesis will be completed. This will be provided in the text and tables to summarise and explain the characteristics and findings of the studies.

For peer review only

Missing data

It is anticipated that rates of attrition and missing data for outcome measurements may be relatively high, and that studies will adopt a variety of methods for handling missing data, including using only complete cases, using all observed data, multiple imputation, baseline observation carried forward, and last observation carried forward. The details of the missing data and data analysis approach will be described in the review. Our main analyses will use whichever analysis is reported in the paper. Where multiple approaches are reported, preference will be given to multiple imputation and all observed data methods. We will conduct a sensitivity analysis to evaluate whether the approach to missing data impacts on the primary outcome.

Confidence in cumulative estimate

The quality of evidence will be assessed using the appropriate CASP checklist. This will be used to judge studies for any methodological flaws. This assessment is suitable due to our inclusion of non-RCTs, PCs and case series design studies. Other assessments more appropriate for RCT design studies may misjudge the quality of studies using these designs.

ETHICS AND DISSEMINATION

The potential of third wave CBTs in weight management has been recently recognised, especially for long-term outcomes. However, evidence of their effectiveness on weight is mixed, and previous reviews have a number of limitations; our review will address these weaknesses.

For peer review only

Our review will distinguish between the different therapies and delivery modes, enabling investigation into their comparative effectiveness. A detailed description of the intervention content, duration and delivery mode will be provided and where possible, meta-regression will be used to identify (and/or to adjust for) the source of heterogeneity across the studies. Besides direct treatment effects using standard random effects pairwise meta-analysis, we will also estimate the indirect treatment effects using random-effects network meta-analysis. This will allow us to include data from a range of prospective studies, not only randomised controlled trials.

Ethical approval is not required as no primary data will be collected. This systematic review will follow the Preferred Reporting for Systematic Reviews and Meta-Analyses (PRISMA) checklist.[29] It is planned that this systematic review will be published in a scientific journal, presented at relevant conferences and will be used in the development of a new weight management programme to help adults with overweight and obesity to reduce weight regain following weight loss. The findings of this review will be of interest to health professionals working with adults with overweight or obesity, researchers involved in the development, evaluation and implementation of weight management interventions, and policy makers and those responsible for commissioning weight management services.

For peer review only

AUTHOR CONTRIBUTORS

ALA, AJH and SJG conceived the study, participated in the study design, provided input on methods, participated in the development of the initial search strategy and contributed to the drafting of the manuscript. ERL participated in the study design, provided input on methods, developed the initial search strategy and was

responsible for drafting the manuscript. NI provided input on methods, participated in the preparation of the initial manuscript draft and reviewed drafts. CH contributed to the drafting of the manuscript. All authors critically reviewed the manuscript and approved the final version submitted for publication.

ACKNOWLEDGEMENTS

The authors would like to thank the University of Cambridge School of Clinical Medicine librarian Eleanor Barker for assistance developing the search strategy.

COMPETING INTERESTS

None declared

DATA STATEMENT

Not applicable

FUNDING

This protocol presents independent research funded by the National Institute for Health Research (NIHR) under its Programme Grants for Applied Research Programme (Reference Number RP-PG-0216-20010). The views expressed are those of the authors and not necessarily those of the NHS, the NIHR or the Department of Health. ALA and SJG are supported by the Medical Research Council [MC_UU_12015/4]. SJG is a National Institute for Health Research (NIHR) senior investigator. The University of Cambridge has received salary support in respect of SJG from the NHS in the East of England through the Clinical Academic Reserve.

For peer review only

REFERENCES

1. Kelly T, Yang W, Chen CS, *et al.* Global burden of obesity in 2005 and projections to 2030. *Int J Obes* 2008;32(9):1431-7. doi: 10.1038/ijo.2008.102.

2. Ng M, Fleming T, Robinson M, *et al.* Global, regional, and national prevalence of overweight and obesity in children and adults during 1980-2013: A systematic analysis for the Global Burden of Disease Study 2013. *Lancet* 2014;384(9945):766-81. doi: org/10.1016/S0140-6736(14)60460-8

3. Arnold M, Pandeya N, Byrnes G, *et al.* Global burden of cancer attributable to high body-mass index in 2012: a population-based study. *Lancet Oncol* 2015;16(1):36-46. doi: 10.1016/S1470-2045(14)71123-4

4. Feigin VL, Roth GA, Naghavi M, *et al.* Global burden of stroke and risk factors in 188 countries, during 1990–2013: a systematic analysis for the Global Burden of Disease Study 2013. *Lancet Neurol* 2016;15(9):913-24. doi: 10.1016/S1474-4422(16)30073-4.

5. Luppino FS, de Wit LM, Bouvy, PF, *et al.* Overweight, obesity, and depression: a systematic review and meta-analysis of longitudinal studies. *Arch Gen Psychiatry* 2010;67(3):220-9. doi: 10.1001/archgenpsychiatry.2010.2.

6. Goettler A, Grosse A, Sonntag D. Productivity loss due to overweight and obesity: a systematic review of indirect costs. *BMJ Open* 2017;7:e014632. doi: 10.1136/bmjopen-2016-014632.

7. Wang YC, McPherson K, Marsh T, *et al*. Health and economic burden of the projected obesity trends in the USA and the UK. *Lancet* 2011;378(9793):815-25. doi: 10.1016/S0140-6736(11)60814-3.

For peer review only

8. Withrow D, Alter DA. The economic burden of obesity worldwide: a systematic review of the direct costs of obesity. *Obes Rev* 2011;12(2):131-41. doi: 10.1111/j.1467-789X.2009.00712.x.

9. National Institute of Health and Clinical Excellence. *Obesity : identification , assessment and management.* NICE, 2014.

10. Hartmann-Boyce J, Johns DJ, Jebb SA, *et al.* Behavioural weight management programmes for adults assessed by trials conducted in everyday contexts: systematic review and meta-analysis. *Obes Rev* 2014;15(11):920-32. doi: 10.1111/obr.12220.

11. Barte JCM, Ter Bogt NCW, Bogers RP, *et al.* Maintenance of weight loss after lifestyle interventions for overweight and obesity, a systematic review. *Obes Rev* 2010;11(12):899-906. doi: 10.1111/j.1467-789X.2010.00740.x.

12. Dombrowski SU, Knittle K, Avenell A, *et al.* Long term maintenance of weight loss with non-surgical interventions in obese adults: systematic review and meta-analyses of randomised controlled trials. *BMJ* 2014;348:g2646. doi: 10.1136/bmj.g2646.

13. Teixeira PJ, Carraça E V, Marques MM, *et al.* Successful behavior change in obesity interventions in adults: a systematic review of self-regulation mediators. *BMC Med* 2015;13:84. doi: 10.1186/s12916-015-0323-6.

14. Ohsiek S, Williams M. Psychological factors influencing weight loss maintenance: an integrative literature review. *J Am Acad Nurse Pract* 2011;23(11):592-691. doi: 10.1111/j.1745-7599.2011.00647.x.

15. Teixeira PJ, Silva MN, Coutinho SR, *et al.* Mediators of weight loss and weight

For peer review only

loss maintenance in middle-aged women. *Obesity* 2010;18(4):725-35. doi: 10.1038/oby.2009.281.

16. Hayes SC, Villatte M, Levin M, Hildebrandt M. Open, aware, and active: contextual approaches as an emerging trend in the behavioral and cognitive therapies. *Annu Rev Clin Psychol* 2011;7:141-68. doi: 10.1146/annurev-clinpsy-032210-104449.

17. Hayes SC. Acceptance and commitment therapy, relational frame theory, and the third wave of behavioral and cognitive therapies. *Behav Ther* 2004;35(4):639-665. doi: 10.1016/S0005-7894(04)80013-3.

18. Linardon J, Fairburn CG, Fitzsimmons-Craft EE, *et al.* The empirical status of the third-wave behaviour therapies for the treatment of eating disorders: a systematic review. *Clin Psychol Rev* 2017;58:125-40. doi: 10.1016/j.cpr.2017.10.005.

19. Williams JMG, Kuyken W. Mindfulness-based cognitive therapy: a promising new approach to preventing depressive relapse. *Br J Psychiatry*. 2012;200(5):359-60. doi: 10.1192/bjp.bp.111.104745.

20. Bowen S, Witkiewitz K, Clifasefi SL, *et al.* Relative efficacy of mindfulness-based relapse prevention, standard relapse prevention, and treatment as usual for substance use disorders: a randomized clinical trial. *JAMA Psychiatry* 2014;71(5):547-56. doi: 10.1001/jamapsychiatry.2013.4546.

21. Öst LG. Efficacy of the third wave of behavioral therapies: a systematic review and meta-analysis. *Behav Res Ther* 2008;46(3):296-321. doi: 10.1016/j.brat.2007.12.005.

For peer review only

22. Carrière K, Khoury B, Günak MM, Knäuper B. Mindfulness-based interventions for weight loss: a systematic review and meta-analysis. *Obes Rev* 2018;19(2):164-77. doi: 10.1111/obr.12623.

23. Katterman SN, Kleinman BM, Hood MM, *et al*. Mindfulness meditation as an intervention for binge eating, emotional eating, and weight loss: a systematic review. *Eat Behav* 2014;15(2):197-204. doi: 10.1016/j.eatbeh.2014.01.005.

24. O'Reilly GA, Cook L, Spruijt-Metz D, Black DS. Mindfulness-based interventions for obesity-related eating behaviours: a literature review. *Obes Rev* 2014;15(6):453-61. doi: 10.1111/obr.12156.

25. Rogers JM, Ferrari M, Mosely K, *et al.* Mindfulness-based interventions for adults who are overweight or obese: a meta-analysis of physical and psychological health outcomes. *Obes Rev* 2017;18(1):51-67. doi: 10.1111/obr.12461.

26. Ruffault A, Czernichow S, Hagger MS, *et al.* The effects of mindfulness training on weight-loss and health-related behaviours in adults with overweight and obesity: a systematic review and meta-analysis. *Obes Res Clin Pract* 2017;11(5 Suppl 1):90-111. doi: 10.1016/j.orcp.2016.09.002.

27. Lyzwinski LN, Caffery L, Bambling M, Edirippulige S. A systematic review of electronic mindfulness-based therapeutic interventions for weight, weight-related behaviors, and psychological stress. *Telemed J E Health* 2018;24(3):173-84. doi: 10.1089/tmj.2017.0117.

28. Olson KL, Emery CF. Mindfulness and weight loss: a systematic review. *Psychosom Med* 2015;77(1):59-67. doi: 10.1097/PSY.0000000000000127.

For peer review only

29. Moher D, Liberati A, Tetzlaff J, Altman DG. Systematic Reviews and Meta-Analyses: The PRISMA Statement. *Annu Intern Med* 2009;151(4):264-269. doi: 10.1371/journal.pmed.1000097.

30. Higgins JPT, Green S. Cochrane Handbook for Systematic Reviews of Interventions Version 5.1.0 [updated March 2011]. The Cochrane Collaboration, 2011. Available at: handbook.cochrane.org.

31. Schulz KF, Altman DG, Moher D, et al. CONSORT 2010 Statement: updated guidelines for reporting parallel group randomised trials. *BMJ* 2010;340:c332. doi: 10.1136/bmj.c332.

32. Hoffmann TC, Glasziou PP, Boutron I, et al. Better reporting of interventions: Template for intervention description and replication (TIDieR) checklist and guide. *BMJ* 2014;348:g1687. doi: 10.1136/bmj.g1687.

33. Critical Appraisal Skills Programme. CASP (Cohort Study) Checklist. [online] Available at: https://casp-uk.net/wp-content/uploads/2018/01/CASP-Cohort-Study-Checklist.pdf . Accessed: 04/04/18.

34. Critical Appraisal Skills Programme. CASP (Randomised Controlled Trial) Checklist. [online] Available at: https://casp-uk.net/wp-content/uploads/2018/01/CASP-Randomised-Controlled-Trial-Checklist.pdf. Accessed: 04/04/2018.

For peer review only

Supplementary file: Medline search strategy

1	exp Obesity/
2	exp Overweight/
3	exp Body Weight/
4	exp Body Mass Index/
5	exp Waist Circumference/
6	exp Feeding Behavior/
7	exp Body Weight Changes/
8	exp Caloric Restriction/
9	exp Weight Loss/
10	obes*.mp.
11	(overweight or over-weight).mp.
12	(weight adj3 (body or chang* or loss* or maint* or manag* or control* or reduct*)).mp.
13	(food adj3 (intake or habit*)).mp.
14	(body mass index or bmi).mp.
15	body adj3 mass.mp.
16	(calori* adj3 (restrict* or restrain* or reduc*)).mp.
17	feeding adj3 behavio*.mp.
18	(diet* adj3 (restrict* or restrain* or reduc*)).mp.
19	(waist* adj3 circumferenc*).mp.
20	1 or 2 or 3 or 4 or 5 or 6 or 7 or 8 or 9 or 10 or 11 or 12 or 13 or 14 or 15 or 16 or 17 or 18
21	((3rd or third) adj3 wave).mp.
22	(acceptance* adj3 (commit* or mind* or base* or focus* or intervention* or therap* or treat*)).mp.
23	exp Mindfulness/
24	(mindful* or mind-ful*).mp.
25	(compassion* adj3 (mind* or base* or focus* or intervention* or therap* or treat*)).mp.

For peer review only

26	(behav* adj3 activation).mp.
27	((meta-cognit* or metacognit*) adj3 (mind* or base* or focus* or intervention* or therap* or treat*)).mp.
28	(dialectic* adj3 (behavio* or mind* or base* or focus* or intervention* or therap* or treat*)).mp.
29	(schema* adj3 (mind* or base* or focus* or intervention* or therap* or treat*)).mp.
30	(function* adj3 analyt*).mp.
31	(relation* adj3 frame*).mp.
32	20 or 21 or 22 or 23 or 24 or 25 or 26 or 27 or 28 or 29 or 30
33	20 and 32

Search terms 21 to 31 based upon Linardon et al.'s[18] search strategy. No database search restrictions will be applied.

For peer review only

PRISMA- P 2015 Checklist

Section and topic	Item number	Checklist item	Criteria location within manuscript
ADMINISTRATIVE INFORMATION			
Title			
Identification	1a	Identify the report as a protocol of a systematic review	Page 1
Update	1b	If the protocol is for an update of a previous systematic review, identify as such	N/A
Registration	2	If registered, provide the name of the registry (such as PROSPERO) and registration number	Page 1
Authors			
Contact	3a	Provide name, institutional affiliation, e-mail address of all protocol authors; provide physical mailing address of corresponding author	Page 1
Contributions	3b	Describe contributions of protocol authors and identify the guarantor of the review	Pages 1, 20 & 21
Amendments	4	If the protocol represents an amendment of a previously completed or published protocol, identify as such and list changes; otherwise, state plan for documenting important protocol amendments	N/A
Support			
Sources	5a	Indicate sources of financial or other support for the review	Page 21
Sponsor	5b	Provide name for the review funder and/or sponsor	Page 21
Role of sponsor or funder	5c	Describe roles of funder(s), sponsor(s), and/or institution(s), if any, in developing the protocol	Page 21
INTRODUCTION			
Rationale	6	Describe the rationale for the review in the context of what is already known	Pages 4 - 7
Objectives	7	Provide an explicit statement of the question(s) the review will address with reference to participants, interventions, comparators, and outcomes (PICO)	Pages 7 & 8
METHODS			
Eligibility criteria	8	Specify the study characteristics (such as PICO, study design, setting, time frame) and report characteristics (such as years considered, language, publication status) to be used as criteria for eligibility for the review	Pages 8 - 10
Information sources	9	Describe all intended information sources (such as electronic databases, contact	Pages 10 & 11

For peer review only

		with study authors, trial registers or other grey literature sources) with planned dates of coverage	
Search strategy	10	Present draft of search strategy to be used for at least one electronic database, including planned limits, such that it could be repeated	Supplementary file
Study records			
Data management	11a	Describe the mechanism(s) that will be used to manage records and data throughout the review	Page 12
Selection process	11b	State the process that will be used for selecting studies (such as two independent reviewers) through each phase of the review (that is, screening, eligibility and inclusion in meta-analysis)	Page 12
Data collection process	11c	Describe planned method of extracting data from reports (such as piloting forms, done independently, in duplicate), any processes for obtaining and confirming data from investigators	Page 12 & 13
Data items	12	List and define all variables for which data will be sought (such as PICO items, funding sources), any pre-planned data assumptions and simplifications	Pages 13 & 14
Outcomes and prioritization	13	List and define all outcomes for which data will be sought, including prioritization of main and additional outcomes, with rationale	Page 14 -16
Risk of bias in individual studies	14	Describe anticipated methods for assessing risk of bias of individual studies, including whether this will be done at the outcome or study level, or both; state how this information will be used in data synthesis	Page 16
Data synthesis	15a	Describe criteria under which study data will be quantitatively synthesised	Pages 16 & 17
	15b	If data are appropriate for quantitative synthesis, describe planned summary measures, methods of handling data and methods of combining data from studies, including any planned exploration of consistency (such as I2, Kendall's τ)	Pages 16 -18
	15c	Describe any proposed additional analyses (such as sensitivity or subgroup analyses, meta-regression)	Pages 17 & 18
	15d	If quantitative synthesis is not appropriate, describe the type of summary planned	Page 18
Meta-bias(es)	16	Specify any planned assessment of meta-bias(es) (such as publication bias across studies, selective reporting within studies)	Page 16
Confidence in cumulative evidence	17	Describe how the strength of the body of evidence will be assessed (such as GRADE)	Page 19

For peer review only

BMJ Open

Third-wave cognitive behaviour therapies for weight management: Systematic review and network meta-analysis protocol.

Journal:	*BMJ Open*
Manuscript ID	bmjopen-2018-023425.R1
Article Type:	Protocol
Date Submitted by the Author:	29-May-2018
Complete List of Authors:	Lawlor, Emma; MRC Epidemiology Unit, University of Cambridge<br>Islam, Nazrul; MRC Epidemiology Unit, University of Cambridge<br>Griffin, Simon; The Primary Care Unit, Institute of Public Health<br>Hill, Andrew; University of Leeds, Division of Psychological and Social Medicine, School of Medicine<br>Hughes, Carly; Fakenham Medical Practice; University of East Anglia Faculty of Medicine and Health Sciences<br>Ahern, Amy; MRC Epidemiology Unit, University of Cambridge
**Primary Subject Heading**:	Public health
Secondary Subject Heading:	Public health
Keywords:	Third-wave cognitive behaviour therapy, Weight management, Weight loss, Weight maintenance, Overweight, Obesity

only

SCHOLARONE™
Manuscripts

Third-wave cognitive behaviour therapies for weight management: Systematic review and network meta-analysis protocol.

Emma R Lawlor[1], Nazrul Islam[1], Simon J Griffin[1,2], Andrew J Hill[3], Carly Hughes[4,5], Amy L Ahern[1]

1. MRC Epidemiology Unit, University of Cambridge, Cambridge, England
2. Primary Care Unit, Institute of Public Health, University of Cambridge, Cambridge, England
3. Division of Psychological & Social Medicine, School of Medicine, University of Leeds, Leeds, United Kingdom
4. Fakenham Medical Practice, Norfolk, United Kingdom.
5. University of East Anglia, Norwich Medical School, Norwich, United Kingdom.

Corresponding author: Emma R Lawlor

Address: MRC Epidemiology Unit, University of Cambridge School of Clinical Medicine, Box 285 Institute of Metabolic Science, Cambridge Biomedical Campus, Cambridge, CB2 0QQ, United Kingdom.

Email: emma.lawlor@mrc-epid.cam.ac.uk

Telephone: +44 (0)1223 330315

Fax: +44 (0)1223 330316

Word count: 3840

For peer review only

ABSTRACT

Introduction: Behavioural and cognitive behavioural programmes are commonly used to assist with weight management, but there is considerable scope to improve their effectiveness, particularly in the longer term. Third-wave cognitive behaviour therapies (CBT) have this potential and are increasingly used. This systematic review will assess the effect of third-wave CBTs for weight management on weight, psychological and physical health outcomes in adults with overweight or obesity.

Methods and analysis: The systematic review will be reported according to the Preferred Reporting Items for Systematic Reviews and Meta-analyses guidance. We will include studies of any third-wave CBTs focusing on weight loss or weight maintenance for adults with a body mass index (BMI) ≥25kg/m^2. Eligible study designs will be randomised control trials, non-randomised trials, prospective cohort and case series. Outcomes of interest will be body weight/BMI, psychological and physical health, and adherence. We will search the following databases from inception to 16th January 2018: MEDLINE, CINHAL, EMBASE, Cochrane database (CENTRAL), PsycINFO, AMED, ASSIA and Web of Science. The search strategy will be based on the concepts: 1) third-wave CBTs 2) overweight, obesity or weight management. No restrictions will be applied. We will search reference lists of relevant reviews and included articles. Two independent reviewers will screen articles for eligibility using a two-stage process. Two independent reviewers will extract data, assess risk of bias using RoB 2.0, ROBINS-I or ROBINS-E checklist and assess quality using GRADE. A random-effects network meta-analysis of outcomes, and sub-group analyses and meta-regression will be conducted, where data permit. If not appropriate, a narrative synthesis will be undertaken.

For peer review only

Ethics and dissemination: Ethical approval is not required as no primary data will be collected. The completed systematic review will be disseminated in a peer-reviewed journal, presented at conferences and used to inform the development a weight management programme.

PROSPERO registration: CRD42018088255

Keywords: Third-wave cognitive behaviour therapy, Weight management, Weight loss, Weight maintenance, Overweight, Obesity

ARTICLE SUMMARY

Strengths and limitations of this study

- Different third wave CBT modalities and delivery methods will be distinguished enabling investigation into their comparative effectiveness.
- In addition to the direct treatment effects, indirect treatment effects will be analysed using a random-effects network meta-analysis.
- A comprehensive search strategy will be used with a large number of databases searched, no limitations applied and all prospective study designs included.
- A description of the intervention content, duration and delivery mode will be provided.
- It is anticipated that many papers will not provide sufficient details on all variables of interest and we will be reliant on communication with corresponding authors for additional information.

For peer review only

INTRODUCTION

Overweight and obesity is a major public health challenge, due to its high prevalence[1,2] and associations with reduced physical and psychological health,[3–5] as well as negative social and economic consequences.[6–8] Supporting people with overweight and obesity to achieve and maintain a healthier weight is an international priority. Most treatment approaches for overweight and obesity combine diet and physical activity advice with psychological support to make behavioural changes. Most commonly, this is a standalone treatment, although it is also an accompaniment to surgery or pharmacotherapy.[9]

There are a number of behavioural programmes that are effective for weight management in the short term, but there is substantial room for improvement in reach and effectiveness – particularly in terms of long term outcomes.[10–12] Whilst many people are able to apply behavioural strategies in order to lose weight, it is hard to sustain these in the face of obesogenic physical and social environments, the biological drive to maintain body weight, and the habitual nature of key behaviours.

One way that the effectiveness of behavioural programmes might be improved is to identify new strategies that target psychological processes associated with better long term weight loss and weight loss maintenance. Key amongst these is sustained motivation and the healthy and adaptive self-regulation of eating behaviour and emotions.[13–15] Third-wave cognitive behaviour therapies (CBTs) have been identified as a potentially useful treatment to address these important factors.

Third-wave CBTs

Third-wave CBTs have a number of core components that distinguish these approaches from first wave behaviour therapy and second wave cognitive behaviour

For peer review only

therapy; specifically an emphasis on openness, awareness, and action.[16] Types of third-wave CBTs include dialectical behavioural therapy (DBT), schema therapy (ST), acceptance and commitment therapy (ACT), acceptance-based behavioural treatment (ABBT), mindfulness-based cognitive behavioural treatment (MBCT) or compassion-focussed therapy (CFT).[16–18] These treatments have been associated with adaptive self-regulation and sustained motivation across a number of health domains, for example, in depression and addiction.[19–21]

There are a number of ways in which these treatments could support successful weight management. For example, fostering non-judgemental awareness of thoughts, feelings and behaviour, could enable people to reduce overeating and to limit the influence of internal and external cues. Encouraging patients to experience potentially aversive internal experiences (cravings, anxiety, behavioural fatigue) and to pursue behaviour that is congruent with their goals and values could support them in adhering to a weight management plan – even when weight loss has plateaued. Fostering a compassionate attitude toward the self could help prevent discouragement following minor lapses. Mindfulness and acceptance-based therapies have also been associated with improvements in psychological outcomes related to long term weight management, including self-regulation, dietary restraint, emotional eating, body satisfaction and mood.[22–26]

Evidence to date

Previous systematic reviews have investigated the effect of third-wave CBTs on weight management.[22–28]

Although these reviews have consistently reported improvements for obesity related eating behaviours and psychological outcomes, results for weight loss have been

For peer review only

mixed. Only three reviews of interventions in people with overweight and obesity included a meta-analysis of weight or BMI outcomes.[22,25,26] Rogers et al.'s[25] meta-analysis of eight studies reported a small but significant reduction in BMI, and the 16 studies included in Carrière et al.'s[22] meta-analysis reported a significant moderate effect on weight loss. However, no significant effect was found for BMI in Ruffault et al.'s[26] meta-analysis of nine studies. These differences may be attributable to variation in inclusion criteria and methods across the reviews.

In addition to mixed findings, there are a number of methodological limitations that constrain conclusions on the effectiveness of third-wave CBT for weight management. The majority of reviews utilised a small number of databases,[24–27] lacked a comprehensive search strategy,[26] and conducted their database searches during, or prior to 2016,[23,25–28] meaning that existing reviews will not have captured all relevant research. In addition, there has been an emphasis on mindfulness, with some reviews excluding other therapy types[22,23] and others using the term 'mindfulness' to encompass a range of third-wave CBTs. There has been a general failure in reviews to distinguish between different types of third-wave CBT or different methods of delivery, and detail on intervention content, delivery mode and intensity is lacking. This causes difficulties in assessing the comparative effectiveness of different approaches and limits their potential to inform future studies. Furthermore, little is known regarding participant adherence and attrition as only Olson and Emery[28] provided detail on this outcome. This would provide important insights into intervention fidelity and acceptability.

The present systematic review will provide a more comprehensive review of the range of third-wave CBTs that have been utilised for weight management. It will interrogate a large number of databases and include all prospective study designs,

For peer review only

while acknowledging the strength of randomised controlled trials. Importantly, it will distinguish between different treatment modalities and delivery methods, by pulling both the direct head-to-head comparisons reported in earlier studies, and the indirect and mixed effects within a random-effects network meta-analysis framework. Meta-regression methods will be applied to identify and/or adjust for potential source(s) of heterogeneity. While change in weight is the primary outcome, this review will also capture a range of physical and psychological health outcomes and will summarise data on intervention adherence. This review will provide important information with which to inform the development and refinement of third-wave CBTs for weight management.

Objectives

We aim to evaluate the effectiveness of third-wave CBTs for weight management in adults with overweight or obesity.

Primary objective

- To evaluate the effectiveness of third-wave CBTs for weight loss and weight loss maintenance in adults with overweight and obesity.

Secondary Objectives

- To evaluate the effect of third-wave CBTs for weight loss and weight loss maintenance on psychological and physical health outcomes in adults with overweight and obesity.
- To provide a detailed description of the content, duration, and delivery of interventions.

For peer review only

- To identify the intervention characteristics that are associated with better outcomes and adherence.

METHODS AND ANALYSIS

Eligibility criteria

Studies will be selected according to the criteria outlined below.

Study designs

We will include original research articles, theses and dissertations reporting randomised control trials (RCTs), non-RCTs, prospective cohort (PC) and case series studies that report an outcome measure pre- and post-intervention. No restrictions will be placed upon language, year of publication or publication status.

Participants

We will include studies of community dwelling adult human participants (aged ≥18 years) with overweight or obesity (BMI ≥ 25 kg/m^2). Participants must be seeking assistance with weight loss or weight loss maintenance. No further restrictions will be made on gender, age, recruitment method, or co-morbid conditions.

Interventions

Studies will be included if they evaluated a third-wave CBT for the purpose of weight loss or weight loss maintenance. In terms of defining third-wave CBTs, they must be described as using DBT, ST, ACT, ABBT, MBCT or CFT. As these approaches are sometimes delivered as part of a multi-component intervention, no restriction will be placed on the proportion of the intervention using the technique, although this may

For peer review only

be considered in secondary-analyses. Interventions will be of any duration or delivery mode. There will be no restriction placed on who delivers the intervention. If multiple arms are included in a study, any arm that meets the inclusion criteria will be included in the review. We will exclude studies that do not state that weight management is an aim of the intervention.

Comparators

For the meta-analysis, we will include studies with no comparator i.e., single-arm pre-post studies with no control arms. We will also compare third-wave CBTs to (i) no intervention or minimal intervention, and (ii) other behavioural programme(s), as reported in the studies.

Outcomes

The primary outcomes will be:

- Body weight
- BMI

The secondary outcomes will be:

- Stress
- Anxiety
- Depression
- Meta-cognition
- Eating attitudes
- Eating behaviours
- Body satisfaction

For peer review only

- Quality of life
- Blood pressure
- Lipids
- Glycaemia
- Adherence to treatment

Timing

We will include studies that report one or more of the primary or secondary outcome measures pre- and post-intervention. We will exclude studies in which the follow up measures are less than 3 months from baseline, because of the identified need to find new approaches to improve long-term weight loss and weight loss maintenance.

Setting

No laboratory based interventions will be included. All other settings are eligible for inclusion.

Language

There will be no language restrictions.

Information sources and search strategy

Electronic searches

We will search the following databases: CINHAL, MEDLINE, EMBASE, Cochrane database (CENTRAL), PsycINFO, AMED, ASSIA and Web of Science. No restrictions to the search strategies will be applied, and databases will be searched from inception to the 16th of January 2018. For studies published in a non-English

For peer review only

language, appropriate employees of the university will be contacted to request a translation.

To identify studies for inclusion in this review, detailed search strategies will be developed for each electronic database by ERL, who has previous experience conducting systematic reviews, with input from a medical librarian. Other members of the systematic review team will also be consulted to ensure appropriateness of terminology and that no terms have been overlooked. The search strategy will include a number of key word and subject heading searches relating to the concepts: (1) Third-wave CBTs AND (2) Overweight, obesity or weight management. Searches in the other databases will be based upon the MEDLINE search strategy (see supplementary additional file), with modification when appropriate to take into consideration database-specific terms. Search terms for the third-wave CBTs concept will be based upon a systematic review of third-wave CBTs for eating disorders by Linardon et al.[18]

Other search methods

To enhance literature saturation, we will manually search reference lists of all primary studies identified as eligible for inclusion in the review and previous relevant reviews. Authors of abstracts that were identified in the database searches will be contacted to identify whether the study findings have been published elsewhere or accepted for publication. A bibliography of the included studies will be circulated to the systematic review team to ensure no other studies they are aware of are excluded.

Study records

For peer review only

Data management and selection process

We will import results from the searches into a Microsoft Excel spreadsheet and duplicates will be removed. We will screen the study titles and abstracts to eliminate articles which clearly do not meet the inclusion criteria. There will be an initial piloting of this screening with an identical 10% of articles independently screened by two researchers to ensure consistency. If a high degree of disagreement occurs, the inclusion and exclusion criteria of the studies will be clarified through discussion with a third reviewer. Once piloting is completed, all article titles and abstracts will be independently screened by two researchers. We will obtain full text papers where titles and abstracts are deemed to be relevant or where eligibility is unclear. The obtained full text articles will then be independently screened by two researchers and their eligibility will be discussed to gain consensus. Where necessary, we will seek additional information from study authors to resolve any questions about eligibility. Reasons for exclusion of articles at the full text screening stage will be recorded. A third reviewer will resolve disagreements, if required. Reviewers will not be blinded to authors, institution or journal when screening articles. In the case of multiple articles pertaining to the same study, all articles will be included and then collated to make best use of the data. A Preferred Reporting Items for Systematic Reviews and Meta-Analyses (PRISMA)[29] flow-chart will be presented showing the process of study selection.

Data collection process

For studies that fulfil the inclusion criteria, we will extract data from articles onto a data collection form. This form will be based upon the Cochrane data extraction form,[30] the Consolidated Standards of Reporting Trials (CONSORT) 2010

statement[31] and the Template for Intervention Description and Replication (TIDieR) checklist.[32] This will ensure that an appropriate breadth and depth of detail will be captured. The data extraction form will be piloted on three articles before it is finalised. Data will be independently extracted by two researchers and any discrepancies will be resolved by a third reviewer. Data analysis will be conducted using statistical program Stata version 14.2 (StataCorp. 2015. *Stata Statistical Software: Release 14*. College Station, TX: StataCorp LP.).

Data items

Data to be extracted from the studies will include:

- General information (e.g. study authors, publication year, country, source of funding)
- Study aim
- Population description
- Study characteristics (e.g. study design, randomisation, blinding, allocation concealment)
- Participants (e.g. age, sex, race/ethnicity, socioeconomic status, diagnosed condition(s), recruitment methods, sample size, weight)
- Intervention characteristics (e.g. therapy type/content, mode of delivery, group or individual delivery, dose of intervention, duration of session, setting, profession who delivered the intervention, theoretical framework)
- Comparator intervention characteristics (e.g. therapy type/content, mode of delivery, group or individual delivery, dose of intervention, duration of session, setting, profession who delivered the intervention, theoretical framework)

For peer review only

- Outcomes (e.g. outcome(s) studied, whether self-reported or objectively measured, duration of follow-up, statistical analysis, intervention effect sizes,)
- Adherence and attrition (e.g. total number of participants at baseline and at follow-up measurements, reasons for attrition, attendance, adherence to intervention)

If studies provide data for multiple follow-up time points, data will be extracted for all time points. If multiple arms are included in a study, data from any arm that meets the inclusion criteria will be extracted.

Due to the objective of this study to provide detailed descriptions of the content of the interventions, attempts will be made to contact the corresponding author of the study to retrieve further information not provided in the article, or in related publications. Study authors will also be contacted if there are any uncertainties regarding the study or missing data. If there is no response, authors will receive two email reminders. Authors will be given a two month timeframe to reply to this request for further information.

Outcomes and prioritisation

The primary outcomes of interest are body weight and BMI as the aim of our review is to evaluate the effect of the third-wave CBTs on weight management. Body weight will be reported as kg. BMI will be defined as body weight (kg) divided by height (m) squared, and will be reported as kg/m^2. We will record whether these measurements have been objectively assessed or have been obtained through participant self-report or other means.

For peer review only

Secondary outcomes are stress, anxiety, depression, meta-cognition, eating attitudes, eating behaviours, body satisfaction, quality of life, blood pressure, lipids, glycaemia and adherence to the third-wave CBT.

Psychosocial outcomes that have been associated with successful weight management have been included in order to understand the potential of third wave CBTs to target these potential determinants of longer term weight control. Data for these outcomes will be extracted as reported in the study. It will be noted if a validated instrument has been used.

For blood pressure, data for systolic and diastolic blood pressure will be extracted separately, and it will be reported using the unit mmHg. For lipids, data will be extracted for total cholesterol, low-density lipoproteins (LDL) and high-density lipoproteins (HDL) and reported using the unit mmol/l. If the ratio of total cholesterol to HDL has been presented, if possible, this will converted into the separate outcomes for total cholesterol and HDL, or the study author will be contacted for raw data. Glycaemia may be reported as glycated haemoglobin ($HbA_{1c;}$ mmol/mol or %) or glucose (random, fasting or 2hr; mmol/l). These outcomes are important as they are closely associated with weight and are risk factors for a range of chronic diseases.

Information relevant to adherence to treatment will include number of sessions attended, length and number of times practicing skills, use of resources, and deviations from intervention instructions. Any information regarding participant attrition/retention, reasons for attrition and attendance at sessions will also be collected. This is important as it will enable investigation into the effect of intensity of

For peer review only

interventions on weight management, and also give insight into the acceptability of the intervention.

Risk of bias of individual studies

Two researchers will independently assess the included studies for risk of bias. Any discrepancies will be discussed with a third reviewer to gain consensus. We will pilot this approach with three studies. Risk of bias will be assessed using the Risk of Bias 2.0 (RoB 2.0) tool,[33] the Risk of Bias in Non-randomised studies of Interventions (ROBINS-I) tool[34] or Risk of Bias in Non-randomised Studies of Exposures (ROBINS-E)[35] Choice of tool will be dependent on the study design. Any other potential sources of risk of bias not covered by the tools will be noted. Reviewers will not be blinded to study authors, journal or institution. Results will be presented in a summary table.

Meta-biases

To assess for selective outcome reporting, if a study protocol is available, the outcomes reported in the protocol and the article will be compared. If not available, the methods and results section of the article will be compared to check for any inconsistencies.

Data synthesis

Intervention comparisons: Direct

Standard pairwise random-effects meta-analysis will be conducted where the homogeneity of data and intervention permit, using Stata version 14.2. The head-to-head comparisons will be conducted on the outcome measures reported at specific time-points of follow-up (e.g., 3 months, 6 months, 12 months). Studies that have

For peer review only

outcomes that fall between these time-points will be combined with the closest time-point. Mean differences (for continuous data) and odds ratio (for categorical data), and their 95% confidence intervals will be estimated and reported.

Intervention comparisons: Indirect and mixed

Random-effects network meta-analysis will be conducted to estimate the indirect and mixed effects using the Stata suite of commands for network meta-analysis, along with commands for visualisation and reporting of results.

Sensitivity analyses

Sensitivity analysis will be conducted to see the potential impact on the effect estimates of different study designs (e.g., RCTs, cohort studies) by restricting the analysis to specific study types and/or by excluding one study designs at a time.

In the case of studies with two or more eligible intervention arms, dependent on the similarity of the interventions, results may be combined or be split into different groups. If different measures have been used to assess the same outcome and are sufficiently similar, they may be pooled or harmonised. If the effect measures are reported in different scales, meta-analysis will use standardised mean differences (standardised by the baseline SD value).

Statistical heterogeneity

The statistical heterogeneity (the portion of the variability that cannot be attributed to random error) of the studies will be tested using the I^2 statistic along with its 95% CI. Forest plots showing the overlap of the confidence intervals will also be provided to enable visual inspection. For the network meta-analysis, we will also report a total I^2

For peer review only

statistic, and the heterogeneity variance parameter (τ^2) estimated from the network meta-analysis models.

Analysis of subgroups or subsets

If sufficient data are available, subgroup analysis will compare:

- Type of intervention (e.g. therapy type, duration, delivery, intensity)
- Comparator intervention (e.g. minimal intervention or other behavioural intervention)
- Health condition of participants (e.g. type 2 diabetes)
- Length of follow up (e.g. 3 months, 6 months, 12 months)
- Study design (e.g. RCT, quasi-RCTs, PC, case series)
- Study quality (e.g. risk of bias)

If sufficient data on important covariates are reported in the studies, meta-regression techniques will be applied to identify and/or adjust for potential sources of heterogeneity, if applicable.

Narrative analysis

If meta-analysis is determined not to be appropriate due to substantial heterogeneity of studies and outcome measures that cannot be pooled, a narrative synthesis will be completed. This will be provided in the text and tables to summarise and explain the characteristics and findings of the studies.

Missing data

It is anticipated that rates of attrition and missing data for outcome measurements may be relatively high, and that studies will adopt a variety of methods for handling

For peer review only

missing data, including using only complete cases, using all observed data, multiple imputation, baseline observation carried forward, and last observation carried forward. The details of the missing data and data analysis approach will be described in the review. Our main analyses will use whichever analysis is reported in the paper. Where multiple approaches are reported, preference will be given to multiple imputation and all observed data methods. We will conduct a sensitivity analysis to evaluate whether the approach to missing data impacts on the primary outcome.

Confidence in cumulative estimate

The quality of evidence will be assessed using the Grading of Recommendations Assessment, Development and Evaluation (GRADE) approach.[36] This will be used to judge studies for any methodological flaws. This assessment is suitable due to our inclusion of non-RCTs, PCs and case series design studies. Other assessments more appropriate for RCT design studies may misjudge the quality of studies using these designs.

Patient and public involvement

This study is part of a larger programme of research funded by an NIHR Programme Grant for Applied Research. In the development of this research programme we held a workshop with 22 members of Fakenham Weight Management Service, who identified the need for support with long term weight management and who felt that psychological therapies were particularly important. The research proposal was then reviewed by three members of Fakenham Weight Management Service and the University of Cambridge PPI Panel prior to submission, then reviewed by PPI representatives on the funding panel, and revised in light of feedback. A PPI

For peer review only

representative (Mrs Jennifer Bostock) is a member of our Investigator team for this research programme, we have two PPI representatives on our programme steering committee (Mr Graham Rhodes and Mrs Norma Scullion), and we have established a Patient User Group panel for the programme. Each of these PPI representatives will contribute to the interpretation and dissemination of findings from this review, and the translation of these findings into a new weight loss maintenance programme.

ETHICS AND DISSEMINATION

The potential of third wave CBTs in weight management has been recently recognised, especially for long-term outcomes. However, evidence of their effectiveness on weight is mixed, and previous reviews have a number of limitations; our review will address these weaknesses.

Our review will distinguish between the different therapies and delivery modes, enabling investigation into their comparative effectiveness. A detailed description of the intervention content, duration and delivery mode will be provided and where possible, meta-regression will be used to identify (and/or to adjust for) the source of heterogeneity across the studies. Besides direct treatment effects using standard random effects pairwise meta-analysis, we will also estimate the indirect treatment effects using random-effects network meta-analysis. This will allow us to include data from a range of prospective studies, not only randomised controlled trials.

Ethical approval is not required as no primary data will be collected. This systematic review will follow the Preferred Reporting for Systematic Reviews and Meta-Analyses (PRISMA) checklist.[29] It is planned that this systematic review will be

For peer review only

published in a scientific journal, presented at relevant conferences and will be used in the development of a new weight management programme to help adults with overweight and obesity to reduce weight regain following weight loss. The findings of this review will be of interest to health professionals working with adults with overweight or obesity, researchers involved in the development, evaluation and implementation of weight management interventions, and policy makers and those responsible for commissioning weight management services.

AUTHOR CONTRIBUTORS

ALA, AJH and SJG conceived the study, participated in the study design, provided input on methods, participated in the development of the initial search strategy and contributed to the drafting of the manuscript. ERL participated in the study design, provided input on methods, developed the initial search strategy and was responsible for drafting the manuscript. NI provided input on methods, participated in the preparation of the initial manuscript draft and reviewed drafts. CH contributed to the drafting of the manuscript and advising and recruiting patient and public involvement. All authors critically reviewed the manuscript and approved the final version submitted for publication.

ACKNOWLEDGEMENTS

The authors would like to thank the University of Cambridge School of Clinical Medicine librarian Eleanor Barker for assistance developing the search strategy. We would like to thank the patient and public involvement representatives who contributed to the development of this research: Mrs Jennifer Bostock, Mr Graham

For peer review only

Rhodes, Mrs Norma Scullion, the University of Cambridge PPI panel, and patients of Fakenham Weight Management Service.

COMPETING INTERESTS

None declared

DATA STATEMENT

Not applicable

FUNDING

This protocol presents independent research funded by the National Institute for Health Research (NIHR) under its Programme Grants for Applied Research Programme (Reference Number RP-PG-0216-20010). The views expressed are those of the authors and not necessarily those of the NHS, the NIHR or the Department of Health. ALA and SJG are supported by the Medical Research Council [MC_UU_12015/4]. SJG is a National Institute for Health Research (NIHR) senior investigator. The University of Cambridge has received salary support in respect of SJG from the NHS in the East of England through the Clinical Academic Reserve.

For peer review only

REFERENCES

1. Kelly T, Yang W, Chen CS, Reynolds K, He J. Global burden of obesity in 2005 and projections to 2030. *Int J Obes*. 2008;32(9):1431-1437. doi:10.1038/ijo.2008.102.

2. Ng M, Fleming T, Robinson M, et al. Global, regional, and national prevalence of overweight and obesity in children and adults during 1980-2013: A systematic analysis for the Global Burden of Disease Study 2013. *Lancet*. 2014;384(9945):766-781. doi:10.1016/S0140-6736(14)60460-8.

3. Arnold M, Pandeya N, Byrnes G, et al. NIH Public Access. 2016;16(1):36-46. doi:10.1016/S1470-2045(14)71123-4.Global.

4. Feigin VL, Roth GA, Naghavi M, et al. Global burden of stroke and risk factors in 188 countries, during 1990–2013: a systematic analysis for the Global Burden of Disease Study 2013. *Lancet Neurol*. 2016;15(9):913-924. doi:10.1016/S1474-4422(16)30073-4.

5. Review AS. Overweight, Obesity, and Depression. 2017;67(3):220-229.

6. Goettler A, Grosse A, Sonntag D. Productivity loss due to overweight and obesity: A systematic review of indirect costs. *BMJ Open*. 2017;7(10). doi:10.1136/bmjopen-2016-014632.

7. Wang YC, McPherson K, Marsh T, Gortmaker SL, Brown M. Health and economic burden of the projected obesity trends in the USA and the UK. *Lancet*. 2011;378(9793):815-825. doi:10.1016/S0140-6736(11)60814-3.

8. Withrow D, Alter DA. The economic burden of obesity worldwide: A systematic review of the direct costs of obesity. *Obes Rev*. 2011;12(2):131-141. doi:10.1111/j.1467-789X.2009.00712.x.

9. Obesity : identification , assessment and management. 2017;(November 2014).

For peer review only

10. Hartmann-Boyce J, Johns DJ, Jebb SA, Summerbell C, Aveyard P. Behavioural weight management programmes for adults assessed by trials conducted in everyday contexts: systematic review and meta-analysis. *Obes Rev*. 2014;15(11):920-932. doi:10.1111/obr.12220.

11. Barte JCM, Ter Bogt NCW, Bogers RP, et al. Maintenance of weight loss after lifestyle interventions for overweight and obesity, a systematic review. *Obes Rev*. 2010;11:899-906. doi:10.1111/j.1467-789X.2010.00740.x.

12. Dombrowski S, Knittle K, Avenell a, Araujo-Soares V, Sniehotta F. Long term maintenance of weight loss with non-surgical interventions in obese adults: systematic review and meta-analyses of randomised controlled trials. *Bmj*. 2014;348(May):g2646. doi:10.1136/bmj.g2646.

13. Teixeira PJ, Carraça E V, Marques MM, et al. Successful behavior change in obesity interventions in adults: a systematic review of self-regulation mediators. *BMC Med*. 2015;13(1):84. doi:10.1186/s12916-015-0323-6.

14. Ohsiek S, Williams M. Psychological factors influencing weight loss maintenance: An integrative literature review. *J Am Acad Nurse Pract*. 2011;23(11):592-691.

15. Teixeira PJ, Silva MN, Coutinho SR, et al. Mediators of weight loss and weight loss maintenance in middle-aged women. *Obesity (Silver Spring)*. 2010;18(4):725-735. doi:10.1038/oby.2009.281.

16. Hayes SC, Villatte M, Levin M, Hildebrandt M. Open, Aware, and Active: Contextual Approaches as an Emerging Trend in the Behavioral and Cognitive Therapies. *Annu Rev Clin Psychol*. 2011;7(1):141-168. doi:10.1146/annurev-

For peer review only

clinpsy-032210-104449.

17. Hayes SC. Acceptance and commitment therapy, relational frame theory, and the third wave of behavioral and cognitive therapies. *Behav Ther*. 2004;35(4):639-665. doi:10.1016/S0005-7894(04)80013-3.

18. Linardon J, Fairburn CG, Fitzsimmons-Craft EE, Wilfley DE, Brennan L. The empirical status of the third-wave behaviour therapies for the treatment of eating disorders: A systematic review. *Clin Psychol Rev*. 2017;58:125-140. doi:10.1016/j.cpr.2017.10.005.

19. Williams JMG, Kuyken W. Mindfulness-based cognitive therapy: a promising new approach to preventing depressive relapse. *Br J Psychiatry*. 2012;200:359-360. doi:10.1192/bjp.bp.111.104745.

20. Bowen S, Witkiewitz K, Clifasefi SL, et al. Relative efficacy of mindfulness-based relapse prevention, standard relapse prevention, and treatment as usual for substance use disorders: a randomized clinical trial. *JAMA psychiatry*. 2014;71:547-556. doi:10.1001/jamapsychiatry.2013.4546.

21. Öst LG. Efficacy of the third wave of behavioral therapies: A systematic review and meta-analysis. *Behav Res Ther*. 2008;46(3):296-321. doi:10.1016/j.brat.2007.12.005.

22. Carrière K, Khoury B, Günak MM, Knäuper B. Mindfulness-based interventions for weight loss: a systematic review and meta-analysis. *Obes Rev*. 2018;19(2):164-177. doi:10.1111/obr.12623.

23. Katterman SN, Kleinman BM, Hood MM, Nackers LM, Corsica JA. Mindfulness meditation as an intervention for binge eating, emotional eating, and weight

For peer review only

loss: A systematic review. *Eat Behav*. 2014;15(2):197-204. doi:10.1016/j.eatbeh.2014.01.005.

24. O'Reilly GA, Cook L, Spruijt-Metz D, Black DS. Mindfulness-based interventions for obesity-related eating behaviours: A literature review. *Obes Rev*. 2014;15(6):453-461. doi:10.1111/obr.12156.

25. Rogers JM, Ferrari M, Mosely K, Lang CP, Brennan L. Mindfulness-based interventions for adults who are overweight or obese: a meta-analysis of physical and psychological health outcomes. *Obes Rev*. 2017;18(1):51-67. doi:10.1111/obr.12461.

26. Ruffault A, Czernichow S, Hagger MS, et al. The effects of mindfulness training on weight-loss and health-related behaviours in adults with overweight and obesity: A systematic review and meta-analysis. *Obes Res Clin Pract*. 2017;11(5):90-111. doi:10.1016/j.orcp.2016.09.002.

27. Lyzwinski LN, Caffery L, Bambling M, Edirippulige S. A Systematic Review of Electronic Mindfulness-Based Therapeutic Interventions for Weight, Weight-Related Behaviors, and Psychological Stress. *Telemed e-Health*. 2017;(September):tmj.2017.0117. doi:10.1089/tmj.2017.0117.

28. Olson KL, Emery CF. Mindfulness and weight loss: A systematic review. *Psychosom Med*. 2015;77(1):59-67. doi:10.1097/PSY.0000000000000127.

29. Moher D, Liberati A, Tetzlaff J, Altman DG. Systematic Reviews and Meta-Analyses: The PRISMA Statement. *Annu Intern Med*. 2009;151(4):264-269. doi:10.1371/journal.pmed1000097.

30. The Cochrane Public Health Group. Cochrane Public Health Group Data

For peer review only

Extraction and Assessment Template. 2011;(November 2011):1-22.

31. Schulz KF, Altman DG, Moher D, et al. CONSORT 2010 Statement: updated guidelines for reporting parallel group randomised trials. *BMC Med*. 2010;8(1):18. doi:10.1186/1741-7015-8-18.

32. Hoffmann TC, Glasziou PP, Boutron I, et al. Better reporting of interventions: Template for intervention description and replication (TIDieR) checklist and guide. *BMJ*. 2014;348(March):1-12. doi:10.1136/bmj.g1687.

33. Higgins JPT, Savović J, Page MJ, Sterne JAC. Revised Cochrane risk of bias tool for randomized trials (RoB 2.0). 2016. doi:file://fs-home/home$/erl42/home/SYS%20REVIEWS%20OF%20THIRDWAVE/RoB2-0_indiv_main_guidance.pdf

34. Sterne JAC, Hernán MA, Reeves BC, et al. ROBINS-I : a tool for assessing risk of bias in non-randomised studies of interventions. *BMJ*. 2016; 355:i4919. doi:10.1136/bmj.i4919.

35. Morgan R, Sterne J, Higgins J, et al. A new instrument to assess Risk of Bias in Non-randomised Studies of Exposures (ROBINS-E): Application to studies of environmental exposure. Abstracts of the Global Evidence Summit, Cape Town, South Africa. *Cochrane Database of Systematic Reviews* 2017;(9 Suppl 1). doi:org/10.1002/14651858.CD201702.

36. Guyatt GH, Oxman AD, Vist GE, Kunz R, Falck-Ytter Y, Alonso-Coello P, et al. GRADE: an emerging consensus on rating quality of evidence and strength of recommendations. *BMJ (Clinical research ed)*. 2008;336(7650):924-6. doi: https://doi.org/10.1136/bmj.39489.470347.AD

For peer review only

For peer review only

Supplementary file: Medline search strategy

1	exp Obesity/
2	exp Overweight/
3	exp Body Weight/
4	exp Body Mass Index/
5	exp Waist Circumference/
6	exp Feeding Behavior/
7	exp Body Weight Changes/
8	exp Caloric Restriction/
9	exp Weight Loss/
10	obes*.mp.
11	(overweight or over-weight).mp.
12	(weight adj3 (body or chang* or loss* or maint* or manag* or control* or reduct*)).mp.
13	(food adj3 (intake or habit*)).mp.
14	(body mass index or bmi).mp.
15	body adj3 mass.mp.
16	(calori* adj3 (restrict* or restrain* or reduc*)).mp.
17	feeding adj3 behavio*.mp.
18	(diet* adj3 (restrict* or restrain* or reduc*)).mp.
19	(waist* adj3 circumferenc*).mp.
20	1 or 2 or 3 or 4 or 5 or 6 or 7 or 8 or 9 or 10 or 11 or 12 or 13 or 14 or 15 or 16 or 17 or 18
21	((3rd or third) adj3 wave).mp.
22	(acceptance* adj3 (commit* or mind* or base* or focus* or intervention* or therap* or treat*)).mp.
23	exp Mindfulness/
24	(mindful* or mind-ful*).mp.
25	(compassion* adj3 (mind* or base* or focus* or intervention* or therap* or treat*)).mp.

For peer review only

26	(behav* adj3 activation).mp.
27	((meta-cognit* or metacognit*) adj3 (mind* or base* or focus* or intervention* or therap* or treat*)).mp.
28	(dialectic* adj3 (behavio* or mind* or base* or focus* or intervention* or therap* or treat*)).mp.
29	(schema* adj3 (mind* or base* or focus* or intervention* or therap* or treat*)).mp.
30	(function* adj3 analyt*).mp.
31	(relation* adj3 frame*).mp.
32	20 or 21 or 22 or 23 or 24 or 25 or 26 or 27 or 28 or 29 or 30
33	20 and 32

Search terms 21 to 31 based upon Linardon et al.'s[18] search strategy. No database search restrictions will be applied.

For peer review only

PRISMA- P 2015 Checklist

Section and topic	Item number	Checklist item	Criteria location within manuscript
ADMINISTRATIVE INFORMATION			
Title			
Identification	1a	Identify the report as a protocol of a systematic review	Page 1
Update	1b	If the protocol is for an update of a previous systematic review, identify as such	N/A
Registration	2	If registered, provide the name of the registry (such as PROSPERO) and registration number	Page 1
Authors			
Contact	3a	Provide name, institutional affiliation, e-mail address of all protocol authors; provide physical mailing address of corresponding author	Page 1
Contributions	3b	Describe contributions of protocol authors and identify the guarantor of the review	Pages 1, 20 & 21
Amendments	4	If the protocol represents an amendment of a previously completed or published protocol, identify as such and list changes; otherwise, state plan for documenting important protocol amendments	N/A
Support			
Sources	5a	Indicate sources of financial or other support for the review	Page 21
Sponsor	5b	Provide name for the review funder and/or sponsor	Page 21
Role of sponsor or funder	5c	Describe roles of funder(s), sponsor(s), and/or institution(s), if any, in developing the protocol	Page 21
INTRODUCTION			
Rationale	6	Describe the rationale for the review in the context of what is already known	Pages 4 - 7
Objectives	7	Provide an explicit statement of the question(s) the review will address with reference to participants, interventions, comparators, and outcomes (PICO)	Pages 7 & 8
METHODS			
Eligibility criteria	8	Specify the study characteristics (such as PICO, study design, setting, time frame) and report characteristics (such as years considered, language, publication status) to be used as criteria for eligibility for the review	Pages 8 - 10
Information sources	9	Describe all intended information sources (such as electronic databases, contact	Pages 10 & 11

For peer review only

		with study authors, trial registers or other grey literature sources) with planned dates of coverage	
Search strategy	10	Present draft of search strategy to be used for at least one electronic database, including planned limits, such that it could be repeated	Supplementary file
Study records			
Data management	11a	Describe the mechanism(s) that will be used to manage records and data throughout the review	Page 12
Selection process	11b	State the process that will be used for selecting studies (such as two independent reviewers) through each phase of the review (that is, screening, eligibility and inclusion in meta-analysis)	Page 12
Data collection process	11c	Describe planned method of extracting data from reports (such as piloting forms, done independently, in duplicate), any processes for obtaining and confirming data from investigators	Page 12 & 13
Data items	12	List and define all variables for which data will be sought (such as PICO items, funding sources), any pre-planned data assumptions and simplifications	Pages 13 & 14
Outcomes and prioritization	13	List and define all outcomes for which data will be sought, including prioritization of main and additional outcomes, with rationale	Page 14 -16
Risk of bias in individual studies	14	Describe anticipated methods for assessing risk of bias of individual studies, including whether this will be done at the outcome or study level, or both; state how this information will be used in data synthesis	Page 16
Data synthesis	15a	Describe criteria under which study data will be quantitatively synthesised	Pages 16 & 17
	15b	If data are appropriate for quantitative synthesis, describe planned summary measures, methods of handling data and methods of combining data from studies, including any planned exploration of consistency (such as I2, Kendall's τ)	Pages 16 -18
	15c	Describe any proposed additional analyses (such as sensitivity or subgroup analyses, meta-regression)	Pages 17 & 18
	15d	If quantitative synthesis is not appropriate, describe the type of summary planned	Page 18
Meta-bias(es)	16	Specify any planned assessment of meta-bias(es) (such as publication bias across studies, selective reporting within studies)	Page 16
Confidence in cumulative evidence	17	Describe how the strength of the body of evidence will be assessed (such as GRADE)	Page 19

For peer review only
